# Brain metabolism in Alzheimer’s disease: biological mechanisms of exercise

**DOI:** 10.1186/s40035-023-00364-y

**Published:** 2023-06-26

**Authors:** Longfei Xu, Ran Liu, Yingkai Qin, Tianhui Wang

**Affiliations:** 1 Institute of Environmental and Operational Medicine, Academy of Military Medical Sciences, Academy of Military Sciences, Tianjin, 300050 China; 2 Tianjin Key Laboratory of Exercise Physiology & Sports Medicine, Tianjin University of Sport, Tianjin, 301617 China

**Keywords:** Alzheimer’s disease, Exercise, Glucose and lipid metabolism, Aβ metabolism, Iron metabolism, Tau pathology

## Abstract

Alzheimer’s disease (AD) is a major subtype of neurodegenerative dementia caused by long-term interactions and accumulation of multiple adverse factors, accompanied by dysregulation of numerous intracellular signaling and molecular pathways in the brain. At the cellular and molecular levels, the neuronal cellular milieu of the AD brain exhibits metabolic abnormalities, compromised bioenergetics, impaired lipid metabolism, and reduced overall metabolic capacity, which lead to abnormal neural network activity and impaired neuroplasticity, thus accelerating the formation of extracellular senile plaques and intracellular neurofibrillary tangles. The current absence of effective pharmacological therapies for AD points to the urgent need to investigate the benefits of non-pharmacological approaches such as physical exercise. Despite the evidence that regular physical activity can improve metabolic dysfunction in the AD state, inhibit different pathophysiological molecular pathways associated with AD, influence the pathological process of AD, and exert a protective effect, there is no clear consensus on the specific biological and molecular mechanisms underlying the advantages of physical exercise. Here, we review how physical exercise improves crucial molecular pathways and biological processes associated with metabolic disorders in AD, including glucose metabolism, lipid metabolism, Aβ metabolism and transport, iron metabolism and tau pathology. How metabolic states influence brain health is also presented. A better knowledge on the neurophysiological mechanisms by which exercise improves AD metabolism can contribute to the development of novel drugs and improvement of non-pharmacological interventions.

## Introduction

Alzheimer’s disease (AD) is a debilitating and progressive degenerative disorder of the central nervous system characterized by insidious memory and cognitive deterioration. AD has become a major public health concern worldwide and displays a high degree of pathophysiological complexity [1–3]. The prevalence of preclinical and clinical AD is projected to nearly triple by 2060 [4]. Key neuropathological features detected in AD brains include interneuronal “senile plaques” dominated by deposits of misfolded amyloid-β (Aβ) peptides and intraneuronal “neurofibrillary tangles (NFTs)” consisting of abnormally accumulating hyperphosphorylated microtubule-associated tau proteins [5]. These features are accompanied by neuronal atrophy and death [6]. Risk factors for the onset and development of AD range from genetic factors, environmental factors to impaired metabolic activity, resulting in a series of pathological cascades. There is a growing consensus that implementing disease-modifying therapies in early stages of AD (preclinical phase) is the most appropriate window to alter the disease course. With the limited efficacy of pharmacological treatments currently available for AD [7], priorities should be shifted toward prevention of AD via non-pharmacological approaches. Despite the correlations of AD with age-related factors, most cases of AD are linked to lifestyle-related risk factors including physical inactivity, unhealthy diets, poor education, and obesity, among others [8]. Thus, emphasis should be placed on lifestyle modifications such as physical activity (PA) in an attempt to slow or prevent the development and manifestation of AD symptomology.

Increased abnormal neuronal metabolism in the brains of AD patients and animal models leads to cognitive decline and a range of complications. Recent research has highlighted the relationship between AD and systemic metabolic changes such as glucose and oxygen hypometabolism [9], lipid peroxidation (abnormal lipid metabolism) [10], dysregulation of Aβ metabolism and transport [11], and deficient and overloaded biogenic metallic elements [12, 13]. Exercise has long been used to manage and prevent such chronic diseases as AD, type 2 diabetes and cardiovascular disease. In fact, a frequently recommended preventive therapy for cognitive impairment in AD patients is regular physical exercise, a strategy based on improvement of brain health [14]. Physical exercise has been shown to help prevent and mitigate the risk and development of AD by affecting neurogenesis, oxidative stress, inflammation and metabolic health, among other pathways [15, 16]. There is mounting evidence that long-term aerobic exercise induces increased hippocampal glucose utilization [17], reduces cholesterol levels [18], restores Aβ degradation and transport [19], alleviates iron overload [20] and reverses sodium loss [21], thereby improving cognitive impairment in AD mouse models and AD patients and enhancing quality of life of patients. In this review, most of the studies we reviewed utilized aerobic exercise as an intervention. Of these, PA is defined as any physical movement produced by skeletal muscle that requires energy expenditure; and exercise, as a subcategory of PA, is planned, structured, repetitive and purposeful in the sense, with the aim of improving or maintaining one or more components of physical fitness (Tables 1 and 2). In addition, other forms of exercise also play a role. Although physical exercise is considered for prevention and treatment of AD [22], there is no consensus on the molecular mechanisms underlying its effects on metabolic imbalances in AD. In this review, we summarize current findings of the mechanisms underlying the effect of exercise interventions on neuronal cell metabolism in AD, in an attempt to highlight the role of metabolic processes and their potential to be pharmacological targets for future treatment.

## Methodology

We searched the PubMed database for literature published over the last decade, using the following keywords: “exercise” OR “Alzheimer’s disease” OR “metabolism” OR “glucose metabolism” OR “lipid metabolism” OR “Aβ metabolism” OR “biogenic metallic elements metabolism” OR “tau metabolism”. There were no restrictions on the research setting, country or language in which the article was originally published. Studies in humans or animals and in vitro studies were included in this review. Their derivative words as well as the bibliographies of related papers were also screened for possible inclusion.

### Exercise maintains the energy metabolism in AD by regulating BDNF, irisin and other targets

There have been many studies on the mechanisms underlying the effect of exercise on AD, focusing on reduction of Aβ plaques and tau protein tangles. Recent studies have suggested that impaired energy metabolism, particularly glucose hypometabolism in the brain, may exacerbate AD pathology [23]. In the nervous system, the main source of energy is the aerobic metabolism of glucose [24]. The energy demands of the brain are mostly met by glucose as a fuel, which requires glycemic control to support several neuronal processes. AD patients have defects in hippocampal glucose metabolism prior to clinical manifestations. The impaired brain glucose uptake and reduced energy supply to neurons may increase the vulnerability of key brain regions to cognitive impairment and dementia [25]. Longitudinal positron emission tomography-based glucose imaging studies have documented reduced glucose uptake and cerebral blood flow throughout the brain and low regional cerebral glucose metabolic rates in patients with AD and prodromal AD [26]. Moreover, several analyses revealed significant hypometabolism and concomitant lower glucose utilization in patients with AD and amnestic mild cognitive impairment (MCI) compared to age-matched neurologically normal subjects [27, 28]. Thomas et al. [29] used a novel non-invasive magnetic resonance imaging method to quantify the whole-brain oxygen metabolic rate in MCI patients and found a reduced overall cerebral oxygen metabolic rate, which was primarily attributed to a reduced oxygen extraction fraction. Likewise, a large body of literature has confirmed that animal models of AD have disturbed glucose metabolism and severe energy deficits in specific brain regions [30–32]. A study in adults with familial and genetic risk of AD showed that 26 weeks of chronic treadmill walking increased brain glucose metabolism and executive function, accompanied by robust gains in cardio-respiratory fitness (CRF), and the improvements in CRF in turn contributed to enhanced cognitive performance and promoted neuroprotective processes in individuals at risk  of AD [33]. Another study noted that 12 weeks of regular high-intensity interval training combined with treadmill walking enhanced CRF and brain glucose uptake in young and older healthy subjects [34]. A cross-sectional study conducted in cognitively healthy middle-aged adults suggested that PA intensity could be a significant contributor to glucose neuronal uptake [35]. These intervention studies suggest that the benefits of exercise for people at risk of AD may be partly mediated by alterations of brain glucose metabolism, primarily in the form of blocking impaired neuronal bioenergetics and stimulating brain metabolic adaptation.

The metabolic fate of glucose in the brain depends on the cell type and selective expression of metabolic enzymes. At the cellular level, the transport of glucose across the blood-brain barrier (BBB) into the extracellular space and subsequent uptake by neurons and glial cells are mediated by specific glucose transporters (GLUTs). Glucose finally enters mitochondria through the glycolytic pathway where it is metabolized via the tricarboxylic cycle and oxidative phosphorylation. GLUTs are essential for the maintenance of cerebral energy metabolism homeostasis [36]. Several converging lines of evidence indicate that GLUT1 and GLUT3 deficiency in the brains of AD transgenic mouse models and AD patients aggravates AD neuropathology coupled with decreased mitochondrial function [37, 38]. Part of the reason for the decline in glucose utilization may be that Aβ-induced oxidative stress impairs GLUT3 function; therefore, GLUT1 and GLUT3 are critical to cerebral glucose homeostasis. Regular PA has been recommended as beneficial for preventing dementia [39]. Single acute exercise upregulates the amount of GLUT1 in endothelial cells and increases lactate levels in the brains of rats [40]. Moreover, a study confirmed that 4 weeks of regular swimming training increased GLUT1 and GLUT3 proteins in the cerebral cortex and hippocampus of AD mice [41], suggesting that exercise improves glucose hypometabolism in AD. Similarly, another study showed that 3 months of chronic voluntary wheel running increased GLUT5 expression and the proportion of GLUT5-positive microglia in the hippocampus of APPswe/PS1dE9 (APP/PS1) mice [42], implying that promoting microglial glucose metabolism may be one of the mechanisms by which exercise delays AD progression. These results indicate that regular exercise improves glucose levels and glycolytic fluxes in vulnerable brain regions and enhances ATP production, adding to the evidence that exercise can benefit patients with AD and promote energy metabolism in the AD brain.

**Fig. 1:**
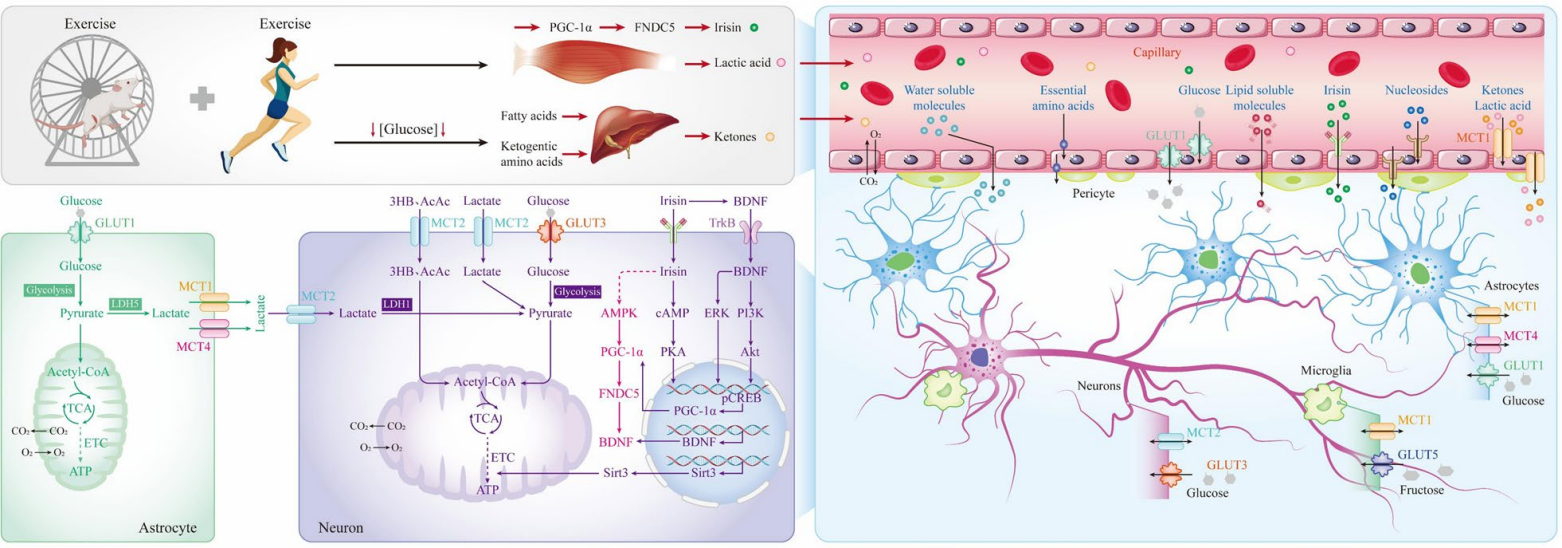
Specific mechanisms by which acute or chronic exercise improves AD glucose metabolism. The major transporter of glucose in brain capillary endothelial cells (ECs) and astrocytes is glucose transporter (GLUT) 1, whereas the major transporters of glucose in neurons and microglia are GLUT3 and GLUT5, respectively. Lactate is capable of crossing the BBB via monocarboxylate transporter (MCT) 1 on ECs to reach the extracellular compartment. Subsequently, lactate enters the cell via MCT1 on astrocytes, oligodendrocytes and microglia for energy metabolism. The entry of lactate into neurons is mediated by MCT2, which also mediates the transport of ketone bodies. In contrast, MCT4 is only expressed in astrocytes and plays fundamental roles in carrying lactate

During the AD process, there are decrements in the functionality of several energy metabolism-related pathways in the brain, including glucose transport, mitochondrial electron transport, and neurotrophic factor signaling [43]. Emerging findings suggest that the brain health is promoted by physical exercise with increased activity in neuronal circuits, including changes in key motor factors and signaling pathways within the AD brain [44, 45]. Consistently, rodent models have demonstrated that exercise can remodel the AD brain for improved metabolism. Molecules that regulate energy metabolism include adenosine 5′-monophosphate-activated protein kinase (AMPK), peroxisome proliferator-activated receptor γ coactivator-1α (PGC-1α), irisin and brain-derived neurotropic factor (BDNF). Irisin is a product from cleavage of fibronectin type III domain-containing protein 5 (FNDC5), and its transcription is regulated by PGC-1α [46]. PGC-1α is activated during contraction of skeletal muscle during exercise, which indirectly up-regulates the expression of FNDC5 in AD models [47] and correspondingly the generation and secretion of irisin, providing further evidence for the potential role of irisin in mediating exercise-induced cognitive benefits in AD models. Irisin can also cross the BBB [48] and trigger the expression of BDNF in neurons [49] to mediate neuroprotection. Irisin acts on neurons through yet unidentified receptors. Irisin stimulates cyclic adenosine monophosphate (cAMP) accumulation, leading to activation of cAMP-dependent protein kinase, phosphorylation of cAMP-response element-binding protein, and BDNF expression to improve memory capacity [50]. The AMPK signaling pathway plays a key role in the regulation of cellular energy homeostasis. Studies have confirmed that abnormal energy metabolism in the AD brain is potentially related to AMPK dysregulation. Upregulation of expression of muscle factor irisin during exercise (treadmill exercise, swimming and sprint training) activates the AMPK signaling pathway [51]. A previous study has shown that regular exercise activates the AMPK/Sirtuin-1 (SIRT1) pathway, which in turn regulates downstream PGC-1α [52]. Many other studies have found that exercise delays cognitive decline primarily through the PGC-1α–FNDC5–BDNF signaling pathway in the AD hippocampus [53–55]. In addition, it has also been shown that physical exercise (3 weeks of chronic treadmill exercise and voluntary wheel running) can increase cerebral glycolysis by enhancing the activities of GLUT1, GLUT3, 6-phosphofructo-2-kinas and lactate dehydrogenase through AMPK activation [56]. Based on the above studies, it can be hypothesized that the exercise-induced cognitive retention and recovery of energy metabolism in AD are mediated through the Irisin/AMPK/PGC-1α/ FNDC5/BDNF signaling (Fig. 1).

BDNF has been recognized as a key regulator of neural circuit development and function and a critical cognitive mediator, as it is highly expressed in brain regions that regulate neuronal differentiation and growth, synaptic formation and plasticity, and higher cognitive processes. BDNF and nerve growth factors are decreased in the brains of AD patients, affecting neuronal survival and plasticity and causing cognitive impairment [57]. A previous study showed that 12 weeks of treadmill exercise increased the phosphorylation levels of extracellular signal-regulated kinase (ERK), phosphatidylinositol-3-kinase (PI3K), protein kinase B (Akt) and glycogen synthase kinase 3β (GSK-3β), accompanied by increased BDNF expression in AD transgenic mice [58]. Previous studies have delineated that BDNF activates its receptor tropomyosin-related kinase B (TrkB) to exert neuroprotective effects. BDNF binding to TrkB promotes activation of the TrkB-mediated signaling pathway including PI3K/Akt and ERK signaling pathways [59], improving cell signal transduction and neurological function. In addition, a study found that multifactor intervention including voluntary wheel exercise and involuntary treadmill running in combination with acousto-optic stimulation, enhanced neurogenesis and neuronal differentiation in the hippocampus, increased protein levels of BDNF, TrkB, and pSer473-Akt in hippocampal lysates, and improved glutamate metabolism, glucose metabolism, and the tricarboxylic acid cycle in a mouse model of AD [60]. Sirtuin-3 (SIRT3) is a mitochondrial deacetylase that is involved in the regulation of mitochondrial energy homeostasis and biogenesis. In the APP/PS1 model, depletion of SIRT3 exacerbates mitochondrial dysfunction [61]. Twenty weeks of treadmill running led to increased SIRT3 protein level and oxidative phosphorylation in the hippocampus of AD mice and attenuated oxidative stress injury [62]. Growing data suggest that exercise-induced delay of AD pathology results from multi-targeting of glucose metabolism, which may also elicit changes in neuronal mitochondrial function in the brain to sustain increased metabolic demands.

Under some specific conditions, neurons can utilize alternative fuels to glucose, namely lactate (glycolysis by-products) and ketone bodies (3-*β*-hydroxybutyrate and acetoacetate) [63]. During high-intensity exercise (with a relative oxygen shortage) and anaerobic exercise, contracting skeletal muscle produces large amounts of lactate, which is released into the bloodstream and transported to the BBB and nerve tissue via specific monocarboxylate transporters (MCTs). Previous studies have reported that MCT2 has an overall higher affinity for many substrates (pyruvate, *L*-lactate, and acetoacetate, etc.) than MCT1 [64]. The specific distribution patterns and differences in affinity for substrates of MCTs in brain cells, as well as the intrinsic metabolic properties of glial cells and neurons can lead astrocytes to absorb glucose from the extracellular fluid and metabolize it into lactate, which is then delivered to neurons [65]. Lactate that shuttles between different cell types in the brain serves as an energy source for neurons to support their physiological activity (lactate shuttle model). There are some controversies on the role of the astrocyte-neuron lactate shuttle compared to direct neuronal uptake of glucose as the primary source of energy for maintaining neuronal physiological function. Accumulating studies show that lactate, a critical energy source and signaling molecule for neurons, contributes to neuron survival and long-term memory formation, while disruption of MCT expression in astrocytes leads to impairment of memory [66–68]. Under normal physiological conditions, the brain lactate concentration is always maintained at a relatively stable level. Impaired lactate efflux leads to abnormally high levels of lactate in the brain, which impairs neurogenesis and causes hippocampal neuronal apoptosis and cognitive decline in AD [69]. Notably, a single acute exercise session up-regulates the levels of MCTs in specific brain regions in Sprague-Dawley rats and  increases brain lactate and β-hydroxybutyrate levels [40]. Furthermore, in a rat model of type 2 diabetes, exercise normalized MCT2 expression level, which was accompanied by increased hippocampal glycogen level and recovery of memory [70]. Based on these results, it is reasonable to speculate that exercise can promote the expression of MCTs in the brains of AD patients, which in turn enhances lactate uptake by neurons to reduce the impairment of neurogenesis caused by abnormally high lactate levels, maintain lactate homeostasis and restore contextual memory. However, more experimental studies are required to determine the specific molecular mechanisms of exercise in humans.

In conditions with limited glucose availability such as during prolonged fasting, low-carbohydrate/high-fat ketogenic diets and prolonged or vigorous exercise, the liver generates ketone bodies 3-*β*-hydroxybutyrate (3HB) and acetoacetate (AcAc) from fatty acid and ketogenic amino acid oxidation, which subsequently enter neurons and glial cells via MCTs to support cellular energy and biosynthetic requirements. Although neuronal glucose metabolism is compromised in AD, the ability of neurons to acquire and utilize 3HB and AcAc is not disrupted [71]. A study confirmed that 3 months of chronic treadmill walking did not improve glucose metabolism in AD patients, but instead increased ketone uptake and utilization [72]. The increased ability of the brain to metabolize ketones could partially be explained by increased cerebral blood flow and/or increased expression of MCTs in the BBB (Fig. 1).

### Exercise accelerates Aβ metabolism by promoting Aβ degradation and efflux in AD

Impaired clearance of Aβ in the brain is a major cause of AD pathogenesis, and clearance of toxic Aβ is essential for maintaining proper intracerebral homeostasis. During AD, impaired function of the Aβ degradation system (the determining condition for Aβ elimination) and the BBB (mediating Aβ efflux clearance) as well as impaired peripheral clearance (systemic elimination of Aβ by the liver or kidneys) results in blockade  of Aβ clearance and subsequent exacerbation of the disease course. Amyloid precursor protein (APP) is a transmembrane protein expressed in a wide range of cell types, including neurons. APP undergoes a constant cycle of trafficking through the endomembrane system: it is generated within the neuronal endoplasmic reticulum and transported to the cell membrane surface via the trans-Golgi network, a process that is precisely regulated [73]. In the amyloidogenic pathway, β-secretase (β-site APP cleaving enzyme1, BACE1) cleaves APP in the extracellular domain to produce the secreted APP ectodomain and the membrane-bound APP carboxyl-terminal fragment. Then γ-secretase cleaves APP into Aβ. In the non-pathogenic pathway, APP is cleaved by α-secretase to release a large ectodomain of APP (sAPP-α), which is subsequently cleaved into non-toxic fragments by γ-secretase. A disintegrin and metalloproteinase 10 (ADAM10) is the main enzymatic component of α-secretase. Previous studies found elevated BACE-1 activity and decreased ADAM-10 expression in AD brains that lead to increased Aβ production and neuronal cell death [74, 75]. In animal models of AD, 5 months of chronic treadmill exercise reduced or prevented increases of BACE1 and Aβ, induced an increase in ADAM17, and attenuated Aβ burdens and astrocytic activation [76]. The clearance of toxic Aβ from the brain is accomplished by major Aβ-degrading enzymes such as neprilysin (NEP) and insulin-degrading enzyme (IDE) [77]. Previous studies have found a beneficial effect of NEP on the degradation of extracellular Aβ plaques. It has been shown that protein levels of NEP and IDE are reduced in the brains of AD models, and that 4 weeks of chronic treadmill running intervention can significantly increase protein levels of both and subsequently reduce Aβ production and deposition [19].

**Fig. 2:**
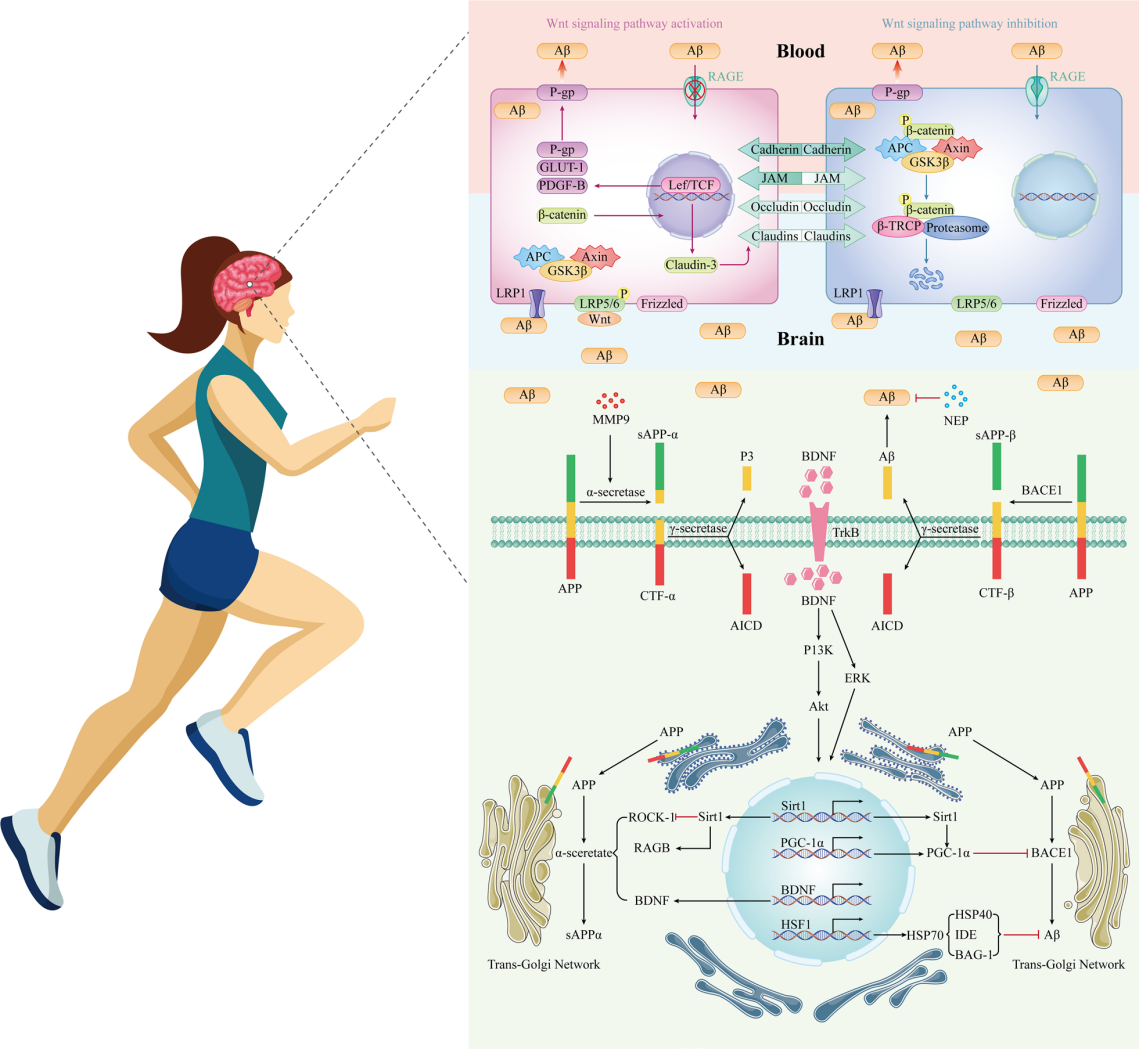
Specific mechanisms by which chronic exercise improves Aβ metabolism. Wnt/FZ forms a ternary cell surface complex with the co-receptor low-density lipoprotein receptor related protein 5/6 (LRP5/6), leading to the recruitment of scatterin and Axin to FZ, which in turn causes activation of the upstream of the Wnt/β-catenin signaling pathway in BBB endothelial cells, and subsequently, this interaction results in a lower phosphorylation of β-catenin in the cytoplasm and stabilization of β-catenin.  β-Catenin enters the nucleus and binds to the lymphatic enhancer factor (Lef)/T-cell factor (TCF) transcriptional factors, leading to the upregulation of claudin-3, GLUT-1, platelet derived growth factor B (PDGF-B) and P-glycoprotein (P-gp), where P-gp transports Aβ from BBB endothelial cells to blood. When Wnt/β-catenin signaling is inhibited in BBB endothelial cells, intracytoplasmic β-catenin is interconnected with the destruction complex, which is mainly composed of colonic adenoma virus (APC), Axin and GSK3β. Disruption of the complex can lead to phosphorylation of β-catenin, followed by separation of the phosphorylated β-catenin from the complex, ubiquitination and degradation of the proteasome, causing a decrease in the level and transcriptional activity of β-catenin in the nucleus, and ultimately dysfunction of the BBB [98]

The specific molecular mechanisms of Aβ clearance by exercise can be summarized as follows. BDNF is the prominent mediator. In vitro cell experiments and animal studies have shown that the exercise-induced elevation of BDNF leads to increased APP cleavage by stimulating α-secretase activity, thereby increasing the production of beneficial sAPPα fragments and decreasing pathogenic Aβ levels [78]. The activity and protein levels of heat shock factor 1 (HSF1), a key regulator for heat shock protein 70 (HSP70) expression, are reduced in AD brain tissues [79], suggesting the existence of a corresponding cascade response to inhibit HSP70 expression and affect protein folding.  In the hippocampus of AD transgenic mice, phosphorylation levels of the PI3K/Akt signaling pathway are reduced [80] and HSP70 expression is inhibited [81], accompanied by increased neuronal loss and apoptosis. Aerobic exercise has been proven to activate the PI3K/Akt signaling pathway and upregulate HSP70 protein level in the brains of AD transgenic mice [58], thereby exerting a neuroprotective effect. Running wheel exercise increases BDNF expression in multiple regions of the brain, and experimental evidence suggests that the BDNF/PI3K/Akt/HSP70 signaling delivered via TrkB mediates many of the beneficial effects of exercise on the brain, and promotes neuronal survival and synaptic growth [82]. In addition, HSP70 combines with auxiliary proteins including heat shock protein 40 (HSP40) [83] and Bcl-2-associated athanogene-1 (BAG-1) to inhibit Aβ aggregation [84], alleviating neurotoxicity of Aβ aggregation. IDE degrades soluble Aβ mainly intracellularly. In addition, HSP70 overexpression significantly increases  the transcriptional and translational levels of IDE in the hippocampus of AD mice [85]. Exercise-induced IDE increase in the hippocampus of AD mice is associated with not only Aβ degradation [19], but also PI3K/Akt pathway activation. Therefore, exercise might activate the BDNF/TrkB/PI3K/Akt signaling pathway in the hippocampus of AD mice, upregulate *HSF1* and *HSP70* expression, increase IDE content, elevate the expression of auxiliary proteins HSP40 and BAG-1, and promote Aβ refolding and degradation (Fig. 2).

SIRT-1 plays an essential role in regulating cellular homeostasis. SIRT-1 regulates Aβ metabolism by modulating APP processing, and SIRT-1 deficiency may lead to reduced levels of ADAM-10 and exacerbate Aβ production in AD patients [86]. On the other hand, SIRT-1 overexpression has been shown to reduce Aβ production [87]. A recent study showed that treadmill exercise increased the expression and activity of SIRT-1 in AD mice and improved physical function [88]. This may explain, at least in part, why exercise enhances the non-amyloid pathway by activating SIRT-1 to block Aβ production. SIRT-1 is known to activate PGC-1α for precise regulation of biological processes. PGC-1α inhibits Aβ production by reducing BACE1 expression and transcription, which may also depend on SIRT-1 signaling [89]. A study demonstrated that SIRT-1 improves AD pathology by upregulating retinoic acid receptor-β (RARβ) to increase ADAM-10 activity [90]. Previous studies have shown that SIRT-1 activation decreases the activity of Rho-related kinase 1 (ROCK-1) in AD mouse neurons, induces ADAM-10 activation and reduces disease onset [91]. Based on the above studies, it could be concluded that exercise increases SIRT-1 levels in AD models and subsequently leads to increased ADAM-10 expression through downregulation of ROCK-1 and upregulation of RARβ. On the other hand, treadmill exercise inhibits BACE1 expression through activation of the SIRT-1/PGC-1α signaling pathway and slows down pathological damage in AD [92].

It is well known that Aβ is transported across the BBB into the brain by receptors for advanced glycation end products (RAGE), and transported out of the brain by low-density lipoprotein receptor-related protein 1 (LRP1). Lower levels of LRP1  expression in endothelial cells around the BBB have been reported in AD patients; inversely, RAGE levels in endothelial cells and neurons have been shown to be elevated in AD patients, leading to accumulation of toxic protein aggregates [93]. Several studies have reported that aerobic exercise can promote Aβ efflux by upregulating LRP1 [19, 94] and downregulating RAGE [76], which contributes to Aβ transport in the brain for peripheral clearance. The Wnt/β-catenin pathway is involved in the proliferation of adult neuronal cells and is regulated by the multifactorial serine/threonine GSK-3β [95]. The presence of highly active GSK-3β in AD patients, which phosphorylates β-catenin and promotes its degradation, disrupts the tight junctions (TJs) of endothelial cells [96]. Studies have shown that long-term moderate exercise activates the hippocampal Wnt/β-catenin signaling pathway in rats, upregulates Wnt expression, inhibits GSK-3β expression and activity, and improves cognitive impairment [97]. We hypothesize that the exercise-activated Wnt/β-catenin signaling pathway could promote synaptic plasticity and neurogenesis by downregulating GSK-3β levels to repair TJs in vascular endothelial cells (e.g., by increasing vascular endothelial cadherin). These studies provide novel and valuable insights into the molecular mechanisms by which exercise reduces Aβ production. More studies are needed to determine their relative contribution.

### Exercise improves lipid metabolism by lowering cholesterol levels to reduce AD neurotoxicity

Lipids are enriched in the CNS and involved in AD pathophysiology. Recent studies have shown a link between lipid metabolism and AD and have identified a number of risk factors for early onset of AD associated with cholesterol metabolism [99]. Recent studies have demonstrated that apolipoprotein E (APOE) and ATP-binding cassette transporter (ABCA1) play a role in lipid metabolism in AD, affecting the production and clearance of Aβ. In the brain, mature neurons are supplied with cholesterol by astrocytes, and cholesterol is transported from neuronal cells throughout the brain to non-neuronal cells via interaction of ApoE with LRP1/low-density lipoprotein family receptors (LDLRs) [100]. The increased intracellular or membrane cholesterol can lead to the increased cleavage of APP and production of Aβ. APOE is mainly synthesized by astrocytes. When APOE is lipidated by free fatty acids, the hydrolytic degradation of Aβ by NEP and IDE is enhanced [101]. ABCA1 is a membrane protein and also a major factor in brain lipid metabolism, which mediates cholesterol efflux to peripheral low-fat or lipid-free apolipoproteins and apolipoprotein A1 (APOA1) [102]. Under normal conditions, cholesterol transported out of the cell via ABCA1 binds to APOA1, which is able to facilitate reverse cholesterol transport. ABCA1 has been used to improve the degradation and clearance of Aβ by increasing the lipidation of APOE [103]. Thus, ABCA1 can decrease Aβ production through the transport of cholesterol to APOE. It has been demonstrated that ABCA1 expression is mainly regulated by liver X receptor (LXR) and retinoid X receptor (RXR) to facilitate the transmembrane transport of cholesterol, and is also involved in Aβ transport and deposition [104]. In vivo and in vitro cellular experimental studies have confirmed that downregulation of ABCA1 expression decreases APOE and increases Aβ load in the AD brain, while upregulation of ABCA1 levels increases APOE expression, promotes APOE lipidation and Aβ clearance, and debilitates the degree of pathology [105]. A study showed that 4 weeks of chronic treadmill exercise may increase ABCA1 expression, stimulate APOE lipidation, and promote Aβ degradation by NEP and IDE to attenuate pathological injury [106]. Another report demonstrated that 5 months of chronic treadmill running down-regulated the level of RXR, increased the expression of LRP1, LDLR, ApoE, LXR and ABCA1, decreased the amount of soluble Aβ in the hippocampus, and improved hippocampal lipid metabolism and plasma lipid levels in APP/PS1 mice [107].

Lipid rafts are dynamic microdomains containing sphingolipids, cholesterol, and phospholipids (particularly phosphatidylcholine) [108]. Such lipids are essential to vesicle trafficking and intracellular transport [109]. During lipid imbalance in AD, alterations in the composition of lipid rafts may affect their physicochemical properties, which in turn alters the local microenvironment and ultimately triggers neurodegeneration [110]. The BACE1 protein is mainly enriched in lipid rafts. When APP is located in lipid rafts, it is more readily cleaved by BACE1 to produce Aβ [111]. Cholesterol is an essential component required for lipid raft generation. A decrease or lack of cholesterol in the intracellular environment inhibits the enzymatic activity of BACE1 [112], suggesting that cholesterol and lipid alignment are key factors regulating BACE1 cleavage of APP. Alterations in cholesterol levels may alter the levels of APP-cleaving enzymes (ADAM10 and BACE1) or the conformation of substrates in lipid rafts, thus affecting Aβ production [113]. Previous in vitro experiments revealed that low cholesterol levels reduced Aβ deposition in the hippocampus and increased α-secretase activity to stimulate non-amyloidogenic cleavage of APP [114]. An observational study by Mann et al. reported that regular exercise exerted beneficial effects on cholesterol level [115], suggesting that exercise may inhibit the amyloid pathway of APP metabolism by decreasing cholesterol levels in AD.

**Fig. 3:**
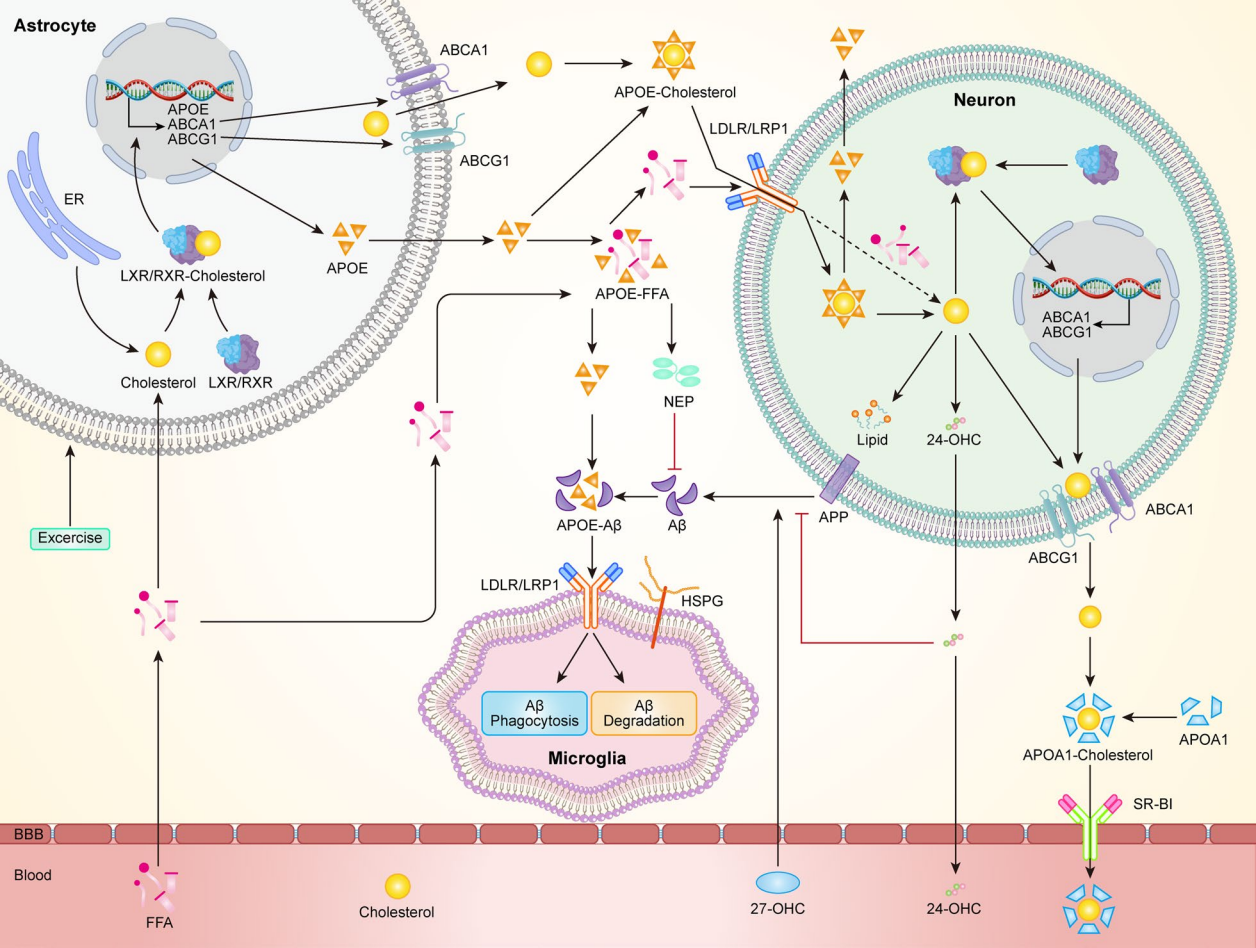
Possible mechanisms by which chronic exercise improves AD lipid metabolism. Free fatty acids (FFAs) cross the BBB and provide an energy substrate for neurons and glial cells. FFAs are converted to cholesterol on the endoplasmic reticulum and bound to LXR/RXR to form LXR/RXR-Cholesterol complexes. Exercise activates the LXR/RXR pathway to increase ABCA1/ABCG1 and APOE expression in astrocytes and mediates cholesterol efflux into the extracellular fluids, promotes APOE lipidation by FFA, and stimulates Aβ degradation by NEP. Lipidated APOE is carried to neurons where the receptor (LDLR/LRP1) removes APOE from lipids and releases FFA into neurons, where it is further involved in neuronal cholesterol metabolism [120]. APOE binds to cholesterol to form APOE-cholesterol particles, which are subsequently mediated by LDLR/LRP1 into neurons and are dissociated. 1% cholesterol is converted into lipid droplets. Most of the cholesterol is catalyzed by enzymes to produce 24-hydroxycholesterol (24-OHC), which subsequently crosses the BBB into the plasma, while plasma 27-OHC flows through the BBB into the brain. A small amount of cholesterol is transported via ABCA1/ABCG1-mediated efflux to the extracellular fluid to form APOA1-Cholesterol particles with APOA1, which are subsequently transported to the blood via receptors. 27-OHC promotes APP cleavage to Aβ, while 24-OHC inhibits the amyloid pathway of Aβ [100]. In addition, Aβ can form an APOE-Aβ complex with APOE, which is attached to the surface of microglia via LDLR/LRP1 and Heparan sulfate proteoglycan (HSPG), and subsequently promotes the uptake and degradation of Aβ via endocytosis of microglia [121]

Flotillin 1 is used as a lipid raft marker, and its abnormal accumulation is associated with the progression of AD. Flotillin 1 recruits APP to lipid rafts to participate in the amyloidogenic pathway. In APP/PS1 mice, cholesterol and Flotillin 1 levels were abnormally elevated, suggesting that lipid raft accumulation in the hippocampus exacerbates the course of AD [116]. Twelve weeks of regular treadmill exercise training significantly reduced the levels of flotillin 1 and cholesterol in AD transgenic mice while upregulating ADAM10 expression and decreasing BACE1 [117]. Thus, exercise may not only reduce Aβ deposition in AD patients, possibly by modulating ADAM10 and BACE1 levels and reducing cholesterol-mediated lipid raft formation, but also reverse cognitive deficits. Dyslipidemia has also been associated with AD [118]. Exercise (4 weeks of chronic involuntary treadmill running combined with swimming) has been shown to improve lipid dysfunction and reduce lipid peroxidation in AD models, and these beneficial effects include improvements in cognitive function and neurogenesis confirmed by experimental analysis [119]. Also, regular exercise increases high-density lipoprotein levels while low-density lipoprotein and triglyceride levels are not changed, exerting systemic lipid-lowering effects [115]. In summary, exercise can regulate abnormal lipid levels in AD, but long-term regular exercise is required to achieve sustained effectiveness (Fig. 3).

### Exercise improves iron metabolism in AD by controlling the transport of iron ions

**Fig. 4:**
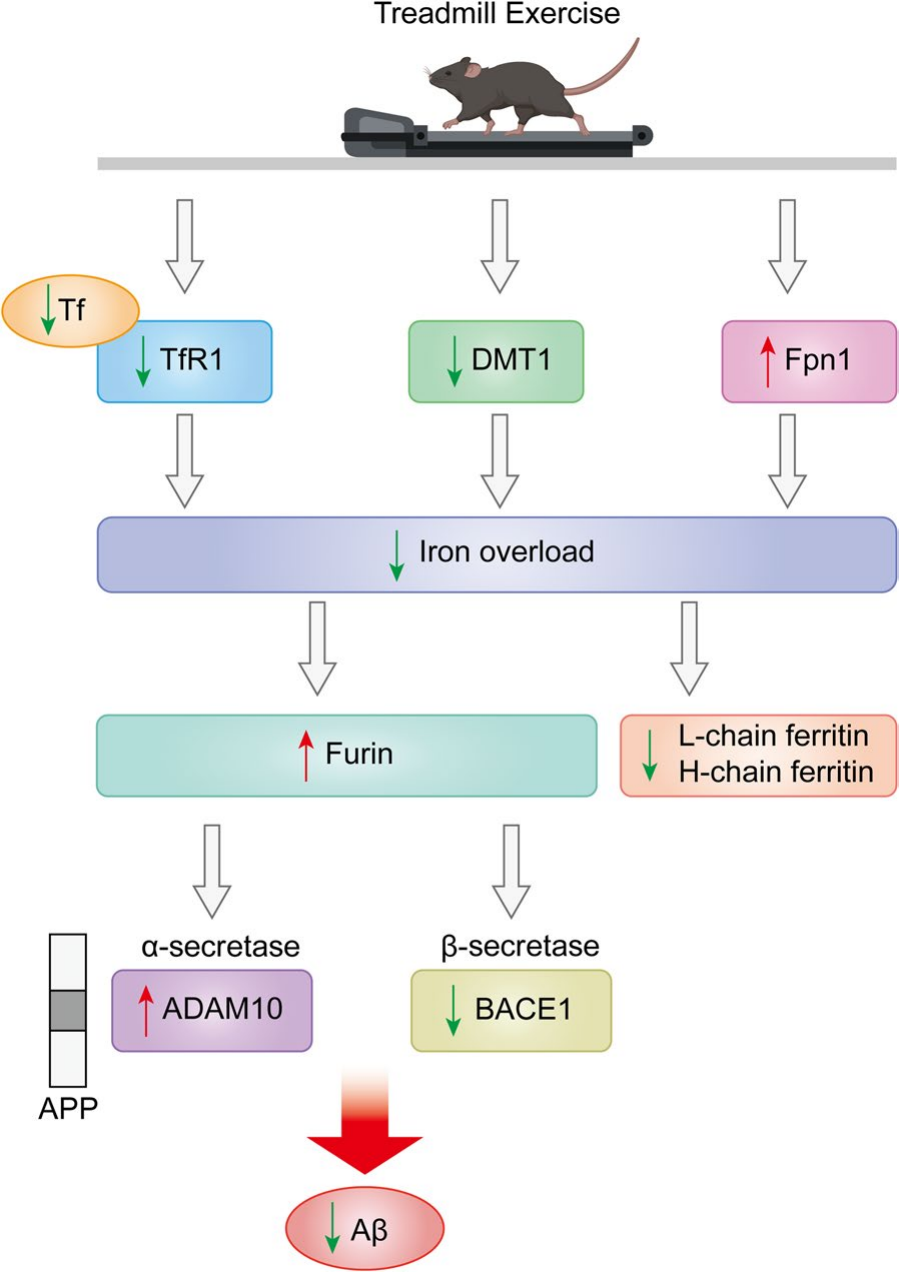
Specific mechanisms by which chronic exercise improves iron metabolism. Exercise induces a synergistic improvement in the balance of iron metabolism in AD brains mainly through regulation of iron transport and related key effector molecules

Age-related diseases including AD are directly associated with metabolic disturbances of biometallic ions (e.g., iron, zinc, copper, and aluminum) in the cortex and hippocampus and are accompanied by neuronal apoptosis, which may be triggered by metal-catalyzed oxidative damage [122]. In pathological AD conditions, substantial evidence points to the involvement of abnormal accumulation of redox-active iron (Fe^2+^) or iron dyshomeostasis that can generate intense oxidative stress, as well as abnormal protein aggregation and ferroptosis, leading to cognitive deterioration [123]. AD may be related to changes in the distribution of iron between different cell types or between different molecular forms (free iron, ferritin, transferrin [Tf], heme, etc.). Inappropriate dramatic increases in ferritin (a major iron storage protein) and iron deposition are strongly associated with the formation of Aβ plaques in the AD hippocampus [124]. Since ferritin promotes the attenuation and sequestration of free iron [125], it may cause elevated levels of labile iron, ultimately leading to increased total iron levels in the brains of AD mice. Cellular transport of iron is regulated by iron uptake transporters (transferrin receptor [TfR] and divalent metal transporter 1) and iron efflux transporters (ferroportin) with the assistance of the ferroxidase ceruloplasmin. Given the association between iron accumulation in AD brains and iron shuttle dysregulation [126], many studies have observed upregulated expression of iron storage protein, ferritin, Tf, TfR, and divalent metal transporter 1 (DMT1) in neurons of AD mice, while ferroportin 1 (Fpn1) and the related protein (ceruloplasmin) are reduced [127–129]. A recent study confirmed that 8 weeks of chronic treadmill training reduced the levels of Tf, TfR and DMT1 and increased the levels of Fpn1 in the motor cortex of AD mice [20]. This suggests that exercise inhibits excessive iron uptake by neurons via down-regulation of iron uptake proteins, and accelerates iron release from neurons via upregulation of iron efflux and iron regulatory proteins, ultimately alleviating iron accumulation and reducing brain iron storage. On the other hand, mitochondria play an important role in iron metabolism. Mitochondria can express DMT1 transporter, which is the major importer of iron for mitochondrial acquisition [130]. Exercise training is known to induce an increase of mitochondrial mass in skeletal muscle [131]. One study demonstrated that 6 months of chronic voluntary wheel running significantly increases DMT1 levels and simultaneously decreases TfR levels in the skeletal muscle of AD mice [132]. In addition, another study found that running wheel exercise reduced iron levels in the plasma and liver, while total iron levels were elevated in tissues with high metabolic activity, such as skeletal muscle, the heart and lung [133]. Based on the above statements, these studies suggest that regular exercise can modulate iron trafficking in AD models by reducing excess iron accumulation in the brain while inducing an increase of mitochondria in skeletal muscle (increasing iron utilization by mitochondria) and redistribution of iron throughout the body (Fig. 4).

Dysregulated iron metabolism and excess iron in AD contribute to amyloidogenesis. Specifically, iron can facilitate Aβ aggregation by modulating the ability of α-secretase and BACE1 to cleave APP [134]. Furin, a ubiquitously expressed proconvertase, modulates systemic iron homeostasis through production of the soluble hemojuvelin, which strongly regulates the processing of α- and β-secretases in AD [135]. At the cellular level in AD patients and animals, excess iron deposition mediates downregulation of *furin* mRNA and protein levels, impairing the α-secretase-dependent processing of APP [136]. For this reason, enhancement of α-secretase activity by reducing iron-mediated damage could delay the harmful effects of Aβ aggregation on the brain. A study has demonstrated that chronic exercise may rectify the functional processing of APP and thus prevent Aβ formation by promoting α-secretase and inhibiting BACE-1, respectively, through low iron-induced enhancement of furin activity in AD mouse model [20], suggesting that exercise, as a means by which to prevent AD-mediated iron imbalance, may be a key modulator in reducing Aβ-induced neuronal death and restoring impaired cognitive function. Another type of key hormone that controls iron balance and regulates iron homeostasis is iron-regulating hormones, which are responsible for negatively regulating iron uptake and efflux from cells. Iron overload in AD patients seems to be triggered by a decrease in iron output due to an increase in hepcidin [137]. Therefore, the reduction of hepcidin in the brain may have a beneficial effect on iron homeostasis in AD patients [138]. The inflammatory state induced by iron load regulates the synthesis of hepcidin, of which interleukin 6 (IL-6) is involved in the process of iron metabolism through hepcidin [126]. IL-6 is increased in the AD brain as a multifunctional cytokine, and high levels of IL-6 can cause memory impairment [139]. In contrast, regular physical exercise attenuates IL-6 expression in the brains of AD mice [140]. Moreover, a study supports that neuroinflammation-induced iron accumulation and hepcidin upregulation in the brain are mediated by the IL-6/signal transducer and activator of transcription 3 (STAT3) molecular pathway [141]. Another study observed a significant decrease in cortical IL-6 and STAT3/Janus Kinase 1(JAK1) levels after voluntary running exercise in AD mice [132]. Therefore, exercise may induce a decrease in hepcidin in the brain through modulation of the IL-6/STAT3/JAK1 pathway, thereby maintaining iron homeostasis and reducing the degree of neurological damage. Exercise-induced changes in hepcidin levels may be paramount in the regulation of cerebral iron metabolism, but the specific regulatory mechanisms need to be further explored.

### Exercise alleviates abnormal tau pathology in AD by mediating bioenergy production

Alterations of tau protein, such as aberrant tau hyperphosphorylation, are a hallmark of AD. Increasing evidence demonstrates that tau pathology overlaps with glucose hypometabolism in the brains of AD patients [142], along with a negative correlation between tau deposition and glucose uptake [143] or aerobic glycolysis [144]. Strikingly, a study has confirmed that pathological tau has a direct impact on mitochondria, inducing neuronal bioenergetic damage and leading to cognitive impairment in AD [145]. Furthermore, another in vitro experiment found that glucose deprivation activates P38 mitogen-activated protein kinase (P38 MAPK), which increases the level of tau phosphorylation at Ser202/Thr205 and Ser404 in cultured N2a mouse adult neuroblastoma cells [146]. This interesting observation has been confirmed by studies in animals expressing human tau (h-tau) [147], demonstrating that aberrant tau hyperphosphorylation and aggregation are mediated by glucose hypometabolism activating the P38 MAPK pathway. Similarly, glucose hypometabolism in the AD brain may activate tau-targeting kinases and thus induce tau lesions, which is interpreted as brain bioenergetic impairment that may be up-stream of tau deposition [148]. The evidence collected here suggests that bioenergetic defects caused by brain glucose underutilisation trigger abnormal tau hyperphosphorylation/aggregation, accelerating tau burdening and paralleling neurological damage and cognitive deficits in AD patients. In turn, pathological tau impairs mitochondrial function, exacerbating the lack of energy production and its own phosphorylation state. Correspondingly, these results also suggest that the reduced bioenergy in AD may be a trigger for the development of tau lesions.

Experimental studies have extensively reported that exercise inhibits the abnormal tau hyperphosphorylation state in the AD brain and exerts neuroprotective effects [149, 150]. Notably, dysregulated glucose metabolism in the AD brain mediates abnormal levels of tau O-GlcNAcylation and consequently its hyperphosphorylation [151]. On the other hand, decreased expressions of GLUT1 and GLUT3 in the brains of AD patients trigger low levels of tau O-GlcNAcylation, resulting in abnormal tau hyperphosphorylation and exacerbating the course of AD[152], whereas four weeks of regular swimming training suppress the decreases of GLUT1 and GLUT3 levels in the brains of AD mice and also downregulate the expressions of Aβ and phosphorylated tau proteins, restoring learning and memory capacity [41]. The above results indicate that exercise alleviates AD symptoms by upregulating GLUT1 and GLUT3 levels in the brain, slowing down impairment of glucose metabolism and improving tau O-GlcNAcylation, thereby inhibiting abnormal tau hyperphosphorylation/deposition. This points to a strong pathological link between energy production caused by glucose metabolism and changes in tau phosphorylation status in the AD brain.

Excessive oxidative stress [153], metabolic disturbances [154] and neuroinflammation [155] in the brain mediate the accumulation of abnormal proteins in the context of AD. Long-term regular endurance exercise acts as an effective physiological regulator to alleviate the pathological state of AD, with multiple neuroprotective effects, and is also essential for maintaining metabolic health. Physical exercise plays a key regulatory role in the enhancement of neuronal activity and neuroprotection by activating signaling molecules including BDNF and elevating the levels of CLUTs in neurons to maintain energy metabolism in the AD brain. In addition, peripheral PGC-1α/FNDC5/Irisin, lactic acid and ketones also contribute to the beneficial effects of exercise on cognitive function and neuronal resilience. Activation of AMPK/SIRT1 and BDNF signaling pathways is known to play a critical role in exercise-related remission of AD pathology [55, 156]. AMPK/SIRT1 and BDNF can directly regulate Aβ production, tau phosphorylation and neurogenesis in the brain by affecting the expression levels of α-, β- and γ-secretases and GSK-3β [78, 157]. Studies have also confirmed that AMPK/SIRT1 and BDNF can regulate glucose uptake by altering insulin and GLUT levels, as well as stimulating PGC-1α-related mitochondrial biogenesis [158, 159]. On the other hand, the mechanisms associated with the ability of exercise to delay AD pathology (Aβ and tau) involve the improvement of glucose metabolism. For instance, IDE not only degrades Aβ in the brain during exercise interventions [76], but also regulates the impaired insulin resistance [160]. In addition to its key role in the translocation of cleared Aβ, LRP1 interacts with insulin receptor β in the brain and regulates insulin signalling and glucose uptake [161]. Four weeks of treadmill training repaired the glucose hypometabolism-related memory damage and abnormal tau hyperphosphorylation in diabetic rats by inhibiting the fork head transcription factor 1/nuclear factor kappa B/pyrin structural domain protein 3 (FOXO1/NF-κB/NLRP3) inflammatory pathway and stimulating the PI3K/Akt insulin pathway [162]. These diverse, interrelated and interacting molecular mechanisms work together to regulate glucose metabolism in the more complex setting of AD, and also highlight that physical exercise has integrated multi-targeting  effects.

Impaired cholesterol homeostasis can cause neurodegenerative diseases. Throughout the clinical phase of AD, high cholesterol levels in the cell membrane lead to high activities of β- and γ-secretases and high production of toxic Aβ peptides [163, 164]. Also, studies have confirmed that the change in cholesterol distribution in the plasma membrane is related to Aβ production [165]. In addition, changes in cholesterol levels can also mediate changes in tau phosphorylation status [166], but the exact molecular mechanisms are unknown and further studies are needed to explain the pathological relationship between cholesterol, Aβ and tau. Based on the current evidence, it is hypothesized that exercise reduces the formation of Aβ peptides and AD pathology by lowering intracellular cholesterol or altering cholesterol distribution. The triggering receptor expressed on myeloid cells 2 (TREM2) is a lipid and lipoprotein receptor on  microglia, and loss-of-function variants of TREM2 lead to impaired cholesterol metabolism and increased incidence of AD [99, 167]. A study found that 3 months of voluntary running inhibited TREM2 shedding, maintained TREM2 protein levels, promoted microglial glucose metabolism in the hippocampus of AD mice, and delayed the disease process [42]. This study, however, did not further explore the changes in lipid levels. Therefore, future studies are needed to determine if exercise improves AD lipid metabolism by affecting TREM2 levels.

Steady-state Aβ levels are the result of the balance between its production and clearance. Based on the above studies, the mechanism by which exercise clears Aβ from the AD brain is more complex and may involve many proteins operating in parallel. In general, this is reflected in the fact that exercise decreases BACE1 and increases α-secretase secretion to reduce toxic Aβ production, upregulates NEP or IDE expression to accelerate Aβ proteolytic degradation, as well as elevates LRP1 and downregulates RAGE levels to facilitate Aβ efflux across the BBB through relevant signaling pathways. Ferroptosis is a unique type of non-apoptotic regulated cell death triggered by acute or chronic cellular stress under aberrant metabolic and biochemical processes, ending in overwhelming iron-dependent lipid peroxidation and cellular rupture [168]. 7-Dehydrocholesterol has greater redox activity, is a precursor of cholesterol, and could be a potential modulator of lipid peroxidation and ferroptosis [169], but questions including whether/how exercise affects ferroptosis and iron overload in AD patients by regulating cholesterol metabolism remain to be answered. In addition, age-related defects in brain glucose metabolism appear to be associated with the progression of tau protein pathology and cognitive impairment in AD [148], that is, mitochondrial dysfunction in the AD state causes bioenergetic impairment that exacerbates abnormal tau phosphorylation and aggregation into NFTs. More longitudinal studies are needed to clarify the specific molecular mechanisms by which physical exercise and energy metabolism alter tau protein pathology and to assess the impact of both on the extent of tau O-GlcNAcylation.

Although epidemiological surveys provide a large-scale database, they do not eliminate all bias/confounding factors or provide specific mechanistic details, which reinforces the need for animal and human clinical studies. Chronic treadmill running for 6 months improves cognitive and executive function and provides many benefits for AD patients by increasing brain glucose disposal [170], and there is growing evidence to support their protective effect against AD [33, 72]. Many of the results on the effects of exercise on AD metabolism as discussed in this paper are mainly based on studies obtained in animal models. However, the study duration and the sample size, which are less limiting in animal studies than in human trials, often lead to discrepancies in results; therefore, further large-scale clinical trials in AD patients are still urgently needed. Exercise helps maintain a healthy cardiovascular system, increases blood flow to the brain and promotes efflux of Aβ, which in turn is directly degraded and cleared by the liver and kidneys, thereby reducing the risk of cognitive decline. Future research is needed to investigate and elucidate the role of peripheral organs in exercise interventions of AD metabolism.

### Conclusions and future guidelines

**Fig. 5:**
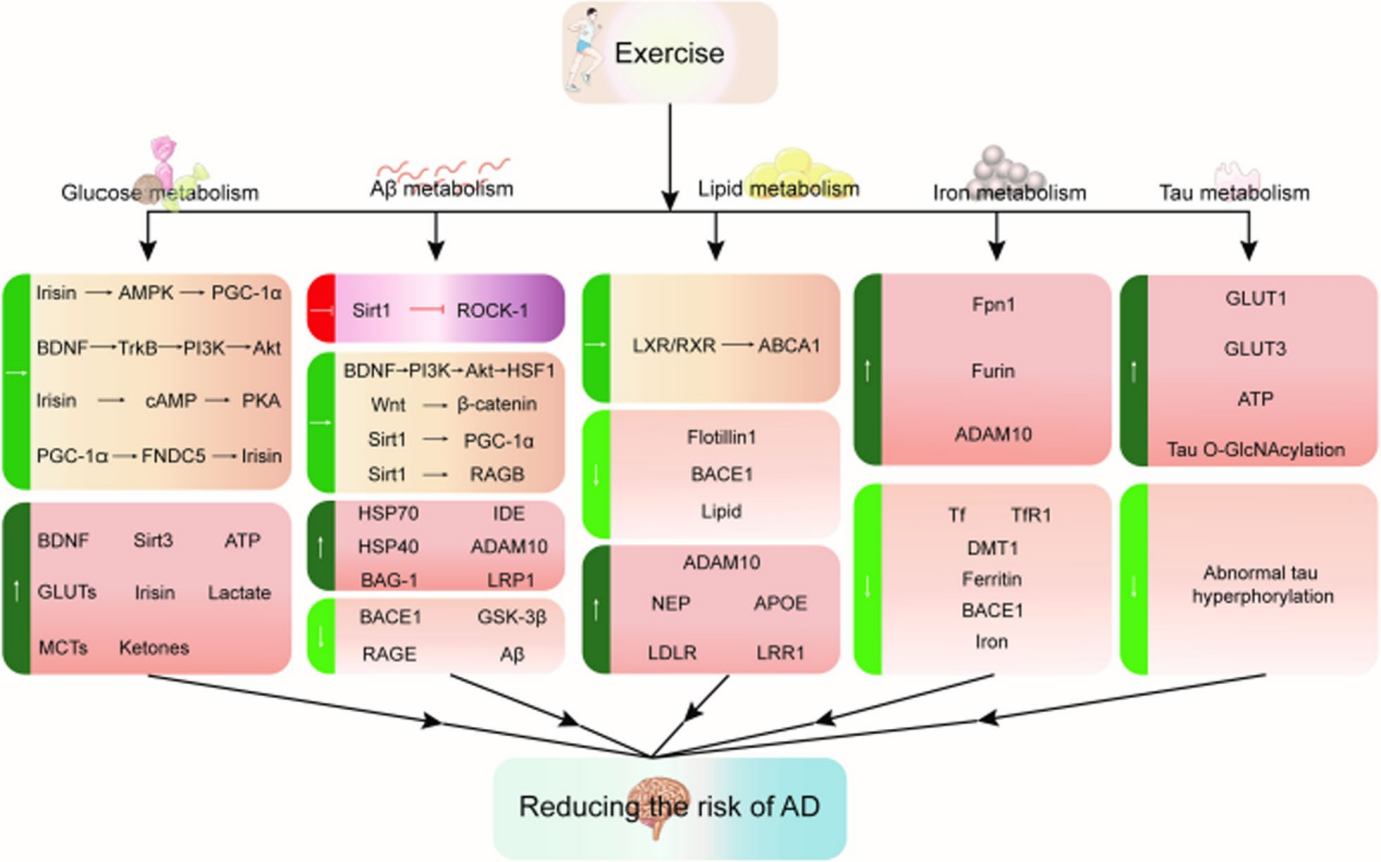
Biometabolic pathways modified by acute or chronic exercise that reduce the risk of AD. Exercise can affect glucose metabolism, Aβ metabolism, lipid metabolism, iron metabolism and tau health, and directly influence AD pathology

To conclude, exercise is a non-invasive way to affect multiple metabolic mechanisms to alter AD pathology. The neuroprotective effects of physical exercise against AD may be due to the synergistic improvement in overall brain metabolism via multiple metabolic targets, ultimately mitigating pathophysiological features and improving cognition (Fig. 5). New insights into the underlying mechanisms linking how exercise biologically affects the metabolic profile of AD and different brain cells can facilitate identification of new and effective targets for AD screening, diagnosis and treatment, as well as the development of promising and tailored combined intervention strategies, effective drug candidates, functional foods and exercise mimetics.

AD pathophysiology is multifaceted and involves a combination of genomic, metabolomic, interactomic and environmental factors. Future translational research on cellular/molecular metabolism and brain health should be actively applied to systems biology to elucidate intercellular and subcellular metabolic pathways, and ultimately reveal brain cell metabolic molecular signatures of AD. Although preclinical studies have proposed potential mechanisms by which exercise can benefit abnormal AD metabolism, there is still a lack of data from human trials to support this. More human studies should be performed in future to unveil the exact biological underpinnings supporting exercise benefits, and to pave the way for personalized physical exercise interventions.

**Table 1:** Effects of physical exercise on brain metabolism in animal models

Model/sex/age	Groups	Exercise protocol	Main effects of glucose/lipid/Aβ/iron/tau metabolism in the brain	Reference
Wistar rats/Male/ 8 weeks	Control groups: intact control group, Aβ injected group, sham injected group Intervention groups: exercise group, Aβ + exercise group	4-week chronic treadmill running/5 days a week/exercise intensity not indicated	NEP, IDE and LRP-1 protein levels were elevated; Aβ levels were reduced	[19]
APP-C105 mice/male/24 months	Control groups: NTg-C group, Tg-C group Intervention groups: NTg-TE group, Tg-TE group	8-week chronic treadmill exercise/5 days a week/70%–85% VO_2max_	Decreased expression of Tf, TfR1, DMT1, ferritin and BACE1; increased expression of Fpn1, Furin and ADAM10; reduced iron overload	[20]
Sprague-Dawley rats/male/10 weeks	Control group: sedentary control group Intervention group: acute exercise group	2-h single acute treadmill exercise/50%–70% VO_2max_/moderate intensity	Increased levels of lactate, β-hydroxybutyrate, MCT1, MCT2, MCT4 and GLUT1 proteins	[40]
APPswe/PS1∆E9 mice/male/6 months	Control groups: WT-NT, AD-NT Intervention groups: WT-T, AD-T	4-week regular swim training/1 h each day, 6 days a week/exercise intensity not indicated	Increased expression of GLUT1 and GLUT3; reduced expression of Aβ and hyperphosphorylated tau proteins; improved glucose metabolism	[41]
APPswe/PS1∆E9 mice/male/10 months	Control groups: WT_Sed, AD_Sed Intervention groups: WT_Run, AD_Run	3-month chronic voluntary wheel running	Increased glucose uptake; increased GLUT5 expression	[42]
Sprague–Dawley rats/male/10–12 weeks	Control group: no exercise Intervention groups: forced treadmill exercise group and voluntary running-wheel exercise group	3-week chronic treadmill exercise/30 min each day, 5 days a week/exercise intensity not indicated; 3-week chronic voluntary wheel running	Enhanced glycolysis; increased levels of GLUT1, GLUT3, phosphofructokinase and lactate dehydrogenase proteins	[56]
Aβ oligomer-induced mice/male/8 weeks	Control groups: saline plus non-intervention group, Aβ oligomer plus non-intervention group Intervention group: Aβ oligomer plus involuntary and voluntary running group	4-week regular involuntary treadmill running/50 min each day, 6 days a week/exercise intensity not indicated; 4-week chronic voluntary wheel running	Increased glutamate metabolism, glucose metabolism and tricarboxylic acid cycle	[60]
APPswe/PS1∆E9 mice/male/5 months	Control groups: SED-Con, SED-APP/PS1 Intervention groups: EXE-Con, EXE-APP/PS1	5-month chronic treadmill exercise/30 min each day, 6 days a week/exercise intensity not indicated	APP, BACE1 and RAGE protein levels were decreased; ADAM10 protein level was increased; Aβ was reduced	[75]
Tg2576 mice/male/ 3 months	Control group: sedentary control group Intervention group: low and high intensity exercise training group	12-week chronic treadmill running/1 h each day, 5 days a week/low and high intensity	Increased levels of NEP, IDE, MMP9, LRP1 and HSP70; increased Aβ degradation and efflux	[93]
Wistar rats/male/ 8 weeks	Control groups: Aβ injection group, sham injection group Intervention groups: Aβ injection + exercise group, sham injection + exercise group	4-week chronic treadmill exercise/5 days a week/mild to moderate intensity	Increased ABCA1 mRNA expression; improved brain lipid metabolism	[105]
APPswe/PS1∆E9 mice/male/3 months	Control group: transgenic control group Intervention groups: transgenic 45%–55% and 60%–70% maximal oxygen uptake exercise groups	5-month chronic treadmill training/30 min each day, 5 days a week/medium and low intensity	RXR and total cholesterol decreased; LXR, LRP1/LDLR, APOE and ABCA1 levels were upregulated; lipid metabolism increased	[106]
APPswe/PS1∆E9 mice/male/3 months	Control groups: WT-NT, AD-NT Intervention groups: WT-T, AD-T	12-week regular treadmill exercise training/45 min each day, 5 days a week/moderate intensity	Decreased cholesterol, BACE1 and flotillin1 levels; decreased number of lipid rafts; increased ADAM10 level	[116]
APPswe/PS1∆E9 mice/male/7 months	Control groups: SED-Con, SED-APP/PS1 Intervention groups: EXE-Con, EXE-APP/PS1	4-week chronic involuntary treadmill running combined with swimming/40 min each day, 6 days a week/mild to moderate intensity	Improved lipid metabolism and amino acid metabolism	[118]
5xFAD transgenic mice/male/6 weeks	Control group: sedentary group Intervention group: voluntarily exercise group	6-month chronic voluntary wheel running	Decreased TfR, DMT1, ferritin, IL-6, STAT3, JAK1 and hepcidin levels; reduced iron overload	[131]

EXE-APP/PS1: Exercise-trained APP/PS1 transgenic mice; EXE-Con: Exercise-trained wild-type control mice; SED-APP/PS1: Sedentary APP/PS1 transgenic mice; SED-Con: Sedentary wild-type control mice; NTg-C: Nontransgenic controls; NTg-TE: Non-transgenic exercise; Tg-C: Transgenic controls; Tg-TE: Transgenic exercise; WT-NT: Wild-type mice group with no exercise; WT-T: Wild-type mice group with regular exercise; AD-NT: APP/PS1 mice group with no exercise; AD-T: APP/PS1 mice group with regular exercise; WT_Sed: Wild-type mice sedentary group; AD_Sed: APP/PS1 mice sedentary group; WT_Run: Wild-type mice running group; AD_Run: APP/PS1 mice running group; VO_2max_: Maximal oxygen consumption

**Table 2:** Effects of physical exercise on brain metabolism in humans

Subjects/age (years)/cognition	Intervention groups	Exercise protocol	Main Effects of glucose /lipid/Aβ/iron/tau metabolism in the brain	Reference
23 adults with a family history of AD/45–80/cognitively normal	Control group: Usual PA group Intervention group: Enhanced PA group	26-week chronic treadmill walking/ 3 times a week/moderate intensity	VO_2_ peak increased; executive function improvement; enhanced glucose metabolism.	[33]
15 (younger) and 12 (older) adults/18–30, 65–80/cognitively normal	Control group: sedentary group Intervention group: HIIT group	12-week regular HIIT combined with treadmill walking/5 days a week/high intensity	VO_2_ peak increased; increased glucose intake.	[34]
4 male and 6 female AD dementia patients/ average age 73/mild dementia	Control group: sedentary control group Intervention group: Walking group	3-month aerobic treadmill training/3 days a week/moderate intensity	Increased uptake and utilisation of ketones; maintained glucose uptake; improved cognition and energy metabolism	[71]
16 male and 17 female AD dementia patients/ average age 70/amnestic MCI	Control group: stretching control group Intervention group: high-intensity aerobic exercise group	6-month chronic treadmill walking/45–60 min each day, 4 days a week/high intensity	Executive function improvement; increased glucose disposal	[169]

VO_2_ peak: peak oxygen consumption; HIIT: high-intensity interval training

## Data Availability

Not Applicable.

## Abbreviations

ABCA1: ATP-binding cassette transporter
AcAc: Acetoacetate
AD: Alzheimer’s disease
ADAM10: A disintegrin and metalloproteinase 10
Aβ: Amyloid-β
Akt: Protein kinase B
AMPK: Adenosine 5′-monophosphate-activated protein kinase
APP: Amyloid precursor protein
APOA1: Apolipoprotein A1
APOE: Apolipoprotein E
BACE1: β-Site APP cleaving enzyme 1
BAG-1: Bcl-2-associated athanogene-1
BBB: Blood-brain barrier
BDNF: Brain-derived neurotropic factor
cAMP: Cyclic adenosine monophosphate
CRF: Cardio-respiratory fitness
DMT1: Divalent metal transporter 1
ERK: Extracellular signal-regulated kinase
FFA: Free fatty acid
FNDC5: Fibronectin type III domain-containing protein 5
Fpn1: Ferroportin 1
GLUT: Glucose transporter
GSK-3β: Glycogen synthase kinase 3β
HIIT: High-intensity interval training
HSF1: Heat shock factor 1
HSP40: Heat shock protein 40
3HB: 3-*β*-Hydroxybutyrate
IDE: Insulin degrading enzyme
IL-6: Interleukin 6
JAK1: Janus kinase 1
LRP-1: Low-density lipoprotein receptor-related protein 1
LXR: Liver X receptor
MCI: Mild cognitive impairment
MCT: Monocarboxylate transporter
NEP: Neprilysin
PGC-1α: Peroxisome proliferator-activated receptor γ coactivator-1α
P-gp: P-glycoprotein
PI3K: Phosphatidylinositol-3-kinase
RAGE: Receptor for advanced glycation end products
RARβ: Retinoic acid receptor-β
ROCK-1: Rho-associated kinase 1
RXR: Retinoid X receptor
SIRT1: Sirtuin-1
Tf: Transferrin
STAT3: Signal transducer and activator of transcription 3
TfR: Transferrin receptor
TJ: Tight junction
TrkB: Tropomyosin-related kinase B
